# Differences in firing patterns along the dorsal-intermediate hippocampal axis in a fixed route during a change in emotional context

**DOI:** 10.3389/fnsys.2025.1632849

**Published:** 2025-11-11

**Authors:** Ryan Troha, Shang Lin (Tommy) Lee, Maya Anam, Sheela Tavakoli, Bailey Morte, Ian H. Stevenson, Etan J. Markus

**Affiliations:** 1 University of Connecticut, Storrs, CT, United States; 2 Emory University, Atlanta, GA, United States

**Keywords:** hippocampus, place cells, aversive learning, approach-avoidance, emotion

## Abstract

The hippocampus plays a prominent role in spatial navigation and memory. However, differences exist along the hippocampus longitudinal axis in function and connectivity. The current study focuses on the dorsal and intermediate subregions of the hippocampus. Single unit CA1 activity was recorded in a fixed route task with a change in emotional valence. We hypothesized the intermediate subregion to show greater changes in general firing activity and place cell remapping in response to emotional change in context compared to the dorsal subregion. Animals were trained to run back and forth for food on a U-shaped maze. In half the trials, animals were presented with a tone which signaled an active shock zone at the apex of the maze. Therefore, animals alternated between “safe” and “unsafe” emotional states, while the spatial configuration of the maze stayed the same. Single-unit activity was recorded and cells were classified by their locations in dorsal hippocampus (DH), anterior intermediate hippocampus (aIH), and posterior intermediate hippocampus (pIH) as well as by spike waveform. Information content was lower and firing rate was higher in the pIH compared to the DH and aIH. A decrease in firing rate was seen in zones close to the shock zone across all three subregions. Contrary to our hypothesis, in well trained animals DH and aIH showed more place cell remapping in response to the tone compared to intermediate regions. Cells in these regions also showed a decrease in firing prior to receiving information regarding the next trial.

## 1 Introduction

Although the hippocampus broadly plays an important role in learning and memory (Jarrard, 1993, 1995; Squire and Knowlton, 1995; Knierim, 2015), its connectivity and function vary along the dorsal-ventral axis (Moser and Moser, 1998; Fanselow and Dong, 2010). Behaviorally, the dorsal hippocampus appears more important for spatial processing (Moser et al., 1995), while more ventral parts of the hippocampus appear to be more strongly linked to the motivational/emotional state of an animal. Lesions to the ventral, but not dorsal, hippocampus reduce anxiety-related behaviors (Kjelstrup et al., 2002), and recent results suggest that ventral regions of the hippocampus are more involved in approach-avoidance conflict evaluation (Schumacher et al., 2018; Yeates et al., 2022; Yoshida et al., 2019; Patterson et al., 2025) and unconditioned fear behavior (Jimenez et al., 2018).

Recent work has begun to tease apart the involvement of distinct hippocampal circuits in different aspects of fear anxiety. Li et al. (2024) show that three distinct populations of interneurons had dissociable effects on different measures of fear and anxiety. All three interneuron types interacted with each other to either inhibit or increase pyramidal cell firing, which was linked to corresponding changes in behavior. Therefore, these interneuron populations mediate specific aspects of fear and anxiety related behavior, placing the ventral hippocampus as a key hub of emotional behavior regulation.

While much research has focused on differences between the dorsal and ventral poles, less is known regarding the transitional region of the intermediate hippocampus. There is evidence for continuous genetic heterogeneity along the full hippocampus longitudinal axis (Thompson et al., 2008). Dong et al. (2009) also indicates a clear distinction along the long axis in gene expression along the CA1 cell layer. The CA1 cell layer can broadly be segregated into dorsal, intermediate, and ventral subregions, with nine CA3 domains correlating with these CA1 subregions. Anatomically, the dorsal hippocampus does not project to the amygdala, while the more posterior part of the intermediate region of the hippocampus projects to the lateral amygdala, and more ventral regions project to the BLA and the posterior basolateral nucleus of the amygdala (BLP) via the intermediate subiculum (Strange et al., 2014; Kishi et al., 2006). In addition, the intermediate subregion does not show direct connectivity with the retrosplenial cortex, unlike the dorsal subregion. The same study showed that the intermediate subregion is also unique from the dorsal subregion in its direct projections to the olfactory cortical areas and medial prefrontal cortical areas (Cenquizca and Swanson, 2007). Intermediate CA1 also has distinct terminal fields in the lateral septum, which then project to the hypothalamus and supramammillary nucleus (Risold and Swanson, 1996). Therefore, the intermediate hippocampus shows a unique genetic profile as well as notable differences in its anatomical connectivity.

Functional differences also distinguish the intermediate subregion from the dorsal and ventral subregions. Place field size increases as one moves more ventrally (Jung et al., 1994) and nonetheless, the intermediate/ventral hippocampus contains some spatial information in addition to its role in emotion and motivation (Lee et al., 2019). Jin and Lee (2021) showed that place cells in the intermediate hippocampus remap after changes in rewards value, while dorsal place cells do not. In addition, inactivation of the ventral-intermediate hippocampus, and not the dorsal hippocampus, impaired the ability of rats to recognize an updated rewards value (Blanquat et al., 2013). Furthermore, lesions of the intermediate, but not dorsal or ventral hippocampus, show impaired rapid place learning (Bast et al., 2009). Therefore, the intermediate hippocampus may rapidly update the representation of an environment. With its role in emotion and motivation, the intermediate hippocampus may be particularly important for processing changes to the emotional or motivational significance of an environment.

Previous studies have shown remapping in the same context after classical fear conditioning and exposure to other aversive stimuli (Moita et al., 2004; Wang et al., 2012; Kim et al., 2015; Schuette et al., 2020). However, these studies focused on the dorsal hippocampus and only recorded activity in a restricted spatial location. In addition, the trajectory of the animal, which is known to induce place cell remapping, was not controlled (Markus et al., 1995; Ferbinteanu and Shapiro, 2003; Schmidt et al., 2012).

Hippocampal units can respond to environmental modifications by changing their firing rates and/or spatial tuning. Global remapping, a change in both the firing rate and field location within an environment, usually occurs after physical or trajectory changes in an environment (Muller et al., 1991; Markus et al., 1995; Ferbinteanu and Shapiro, 2003). Rate remapping, a change in firing rate without a change in place field location (Leutgeb et al., 2005), usually occurs after subtle changes to an environment. Rate remapping has been proposed as a mechanism for representing non-spatial changes to an environment (Allen et al., 2012; Leutgeb et al., 2005; Sanders et al., 2019; Lu et al., 2015).

The current study compared single-unit activity in CA1 along the dorsal-intermediate axis. Animals ran back and forth on a U-shaped maze and, on some trials, were presented with a tone signaling an active shock zone at the apex of the maze. Importantly, the trajectory of animals remained the same and only the emotional context (“safe” vs. “unsafe”) changed. Previously we showed in this task that knowing where to hesitate is hippocampus dependent (Oler et al., 2005), with little remapping in the dorsal hippocampus (Oler et al., 2008) compared to a task with a trajectory change (Markus et al., 1995).

## 2 Materials and methods

### 2.1 Subjects

Subjects consisted of six male F344 rats (Envigo, Indianapolis, IN) aged approximately 4 months at the start of training. Animals underwent surgery, recovery, and post-surgery experimentation between 6 and 12 months of age. Animals were maintained between 80% and 85% of their free-feeding weights throughout the experiment. All experimental protocols were approved by the University of Connecticut IACUC.

### 2.2 Hyperdrive construction

Hyperdrive recording devices contained 16 independently moveable tetrodes. Each tetrode was composed of four polyamide-coated 14 μm nickel-chrome wires (Sandvik) twisted together. Electrodes were gold plated to reduce single channel impedances to approximately 150–500 kΩ at 1 kHz by passing current through the wires while the electrode tips were immersed in a mixture of gold plating (PAS-5355; Sifco) and polyethylene glycol solution (PEG 8000 MW; Sigma-Aldrich) (Ferguson et al., 2009). Prior to surgery, tetrodes were dipped in different colors of fluorescent microsphere solution (FluoSpheres; ThermoFisher F8829).

### 2.3 Pre-surgery behavioral training

**FIGURE 1:**
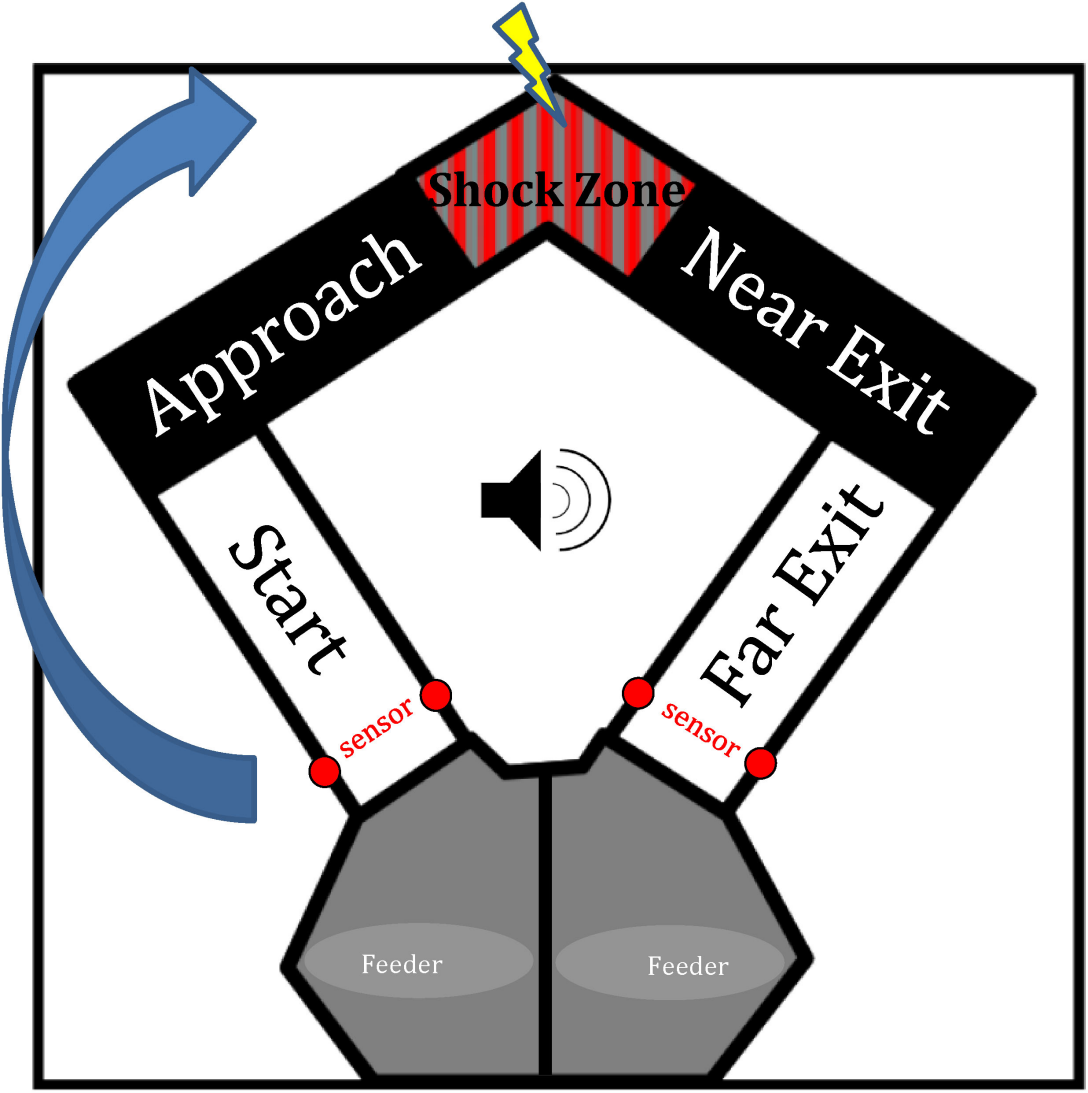
Diagram of the U-shaped maze. The apex of the maze contains a shock zone which would either be active (i.e., tone trials) or inactive (i.e., no-tone trials). The maze was divided into zones in order to analyze behavior and firing activity. The arrow here indicates a trial in which the rat would start in the left feeder zone and finish by entering the right feeder zone. Sensors near feeder zones trigger the start/end of each trial as animals complete a trial by traversing the far exit zone.

Animals were first conditioned to the approach-avoidance behavioral task prior to surgery. Training took place in 30 min daily training sessions. First, animals learned to run back and forth on the U-shaped maze (Figure 1) to obtain food until they completed two consecutive days of 60+ runs (i.e., a run defined as traversing the maze and entering the opposite feeder area). Next, animals completed training sessions where 10% of the trials included the tone and mild current in the shock zone, while the rest of the trials included no tone or shock. Again, animals were trained until they completed two consecutive days of 60+ runs. The current level was titrated to the animal’s performance in the range of 0.15–0.3 mA until animals reached 50% of the trials including the tone and shock, with the order of trial types being determined pseudorandomly. Animals stayed on this training schedule until, over 10 days, paired *t*-tests showed differences in the time spent in either the “start” or “approach” zone between tone-shock and no-tone-shock trials. In addition, animals were required to complete at least 40 trials in each of their last 10 training sessions. During analysis, instances where the animal touched the shock zone and turned around were excluded to ensure that animals were hesitating specifically to the tone and maze location and not in response to the shock stimulus. This ensured that prior to surgery, animals discriminated between trial types, but were still comfortable running through the shock zone to obtain food. Once these criteria were met, animals underwent surgery, recovered for 10 days, and began re-training on the task.

### 2.4 Surgery

Animals were anesthetized with isoflurane prior to undergoing stereotaxic surgery. During induction 3% isoflurane in O_2_ was used, while 1%–3% was used for maintenance throughout surgery. After confirmation of anesthesia, meloxicam (Metacam, 1 mg/kg, s.c.) was injected, each rat was placed in a stereotaxic apparatus with blunt ear bars, ophthalmic ointment was applied on the eyes, and the scalp was shaved and wiped with 2% chlorhexidine, 70% ethanol, and Betadine. A small midline scalp incision was made, and a craniotomy was drilled over the right dorsal hippocampus (−4.7 mm anteroposterior and 3.0 mm mediolateral from bregma) and right ventral hippocampus (−6.8 mm anteroposterior and 5.8 mm mediolateral from bregma) using the rat brain atlas (Paxinos and Watson, 2006). Seven stainless steel screws were secured to the skull. After the dura was carefully removed, the hyperdrive was positioned above the craniotomy. Silicone elastomer (Kwik-sil; WPI) covered the remaining exposed brain within the craniotomy. Dental acrylic (B1334; Ortho-Jet BCA) was applied to the hyperdrive base and screws to anchor the device. After hyperdrive implantation, two additional injections of Meloxicam were administered post-operatively at 24 and 48 h after surgery. Rats received 10 days of post-operative recovery before being food-restricted to 85% of their free feeding weight and behavioral testing. Tetrodes were advanced ∼200 microns per day for the first 7–10 days (or until sharp waves were detected) post-surgery. Once the hippocampus was reached, tetrodes were advanced ∼40 microns per day until the pyramidal cell layer was reached. Tetrodes were only advanced ventrally except in the rare case that a tetrode unexpectedly advanced beyond the pyramidal cell layer.

### 2.5 Post-surgery behavioral training

After recovery and food restriction following surgery, animals were re-trained. Animals began re-training with no tether connected to the hyperdrive and simply ran back and forth for food with no tone/shock trials included. Once animals completed two consecutive sessions with 60+ trials, they then began training with 10% of the trials including a tone and shock. Once animals completed 40+ trials on two consecutive days, the recording tether was then connected. Once animals again reached two consecutive days of 40+ trials with the tether connected, the percentage of shock trials increased gradually until 50% of the trials included no tone or shock, 25% included tone only, and 25% included the tone and shock. The order of these trial types was then changed pseudorandomly across daily experimental sessions. Before recording, animals were placed next to the maze for a 5 min pre- and post-recording session. Between daily recording sessions, tetrodes were advanced throughout the hippocampus to find new cells. An average of 17 recording sessions were collected per animal.

### 2.6 Data acquisition

The electrode interface board (EIB-72-QC-Large; Neuralynx) on the hyperdrive was connected to a multichannel, unity gain headstage (HS-72-QC-LED; Neuralynx). The output of the headstage was conducted via two lightweight tether cables to a digital data acquisition system which then processed the signals (Digital Lynx SX; Neuralynx). Continuously sampled raw broadband data (between 0.1 and 8,000 Hz) was acquired and saved at 32 kHz prior to being processed. The headstage had an array of light-emitting diodes (three blue and three red LEDs) to track the rat’s position and head direction. XY position coordinates of both diode arrays was sampled at 29.97 Hz using a deep learning tracking method (DeepLabCut; Mathis et al., 2018). Speed was then calculated by taking the finite difference between successive position coordinates followed by a low-pass filter (cutoff = 0.25 Hz) to minimize movements and other head-movement related artifacts.

### 2.7 Spike sorting

MountainSort automated spike sorting algorithm was used to bandpass filter (600–6,000 Hz) the raw data, detect spikes, and isolate single-units (Chung et al., 2017). Cluster quality thresholds were applied to only include single units with greater than 95% isolation, less than 3% noise overlap, and greater than 1.5 peak signal-to-noise ratio (SNR). An average of 6.5 cells per tetrode were identified as passing criteria. The isolation metric quantifies how well separated the cluster is from other nearby clusters, the noise overlap metric estimates the fraction of above-threshold events not associated with true firings in a clustered unit, and the peak SNR is defined as the peak absolute amplitude of the average waveform divided by the peak standard deviation. This was followed by manual curation of clusters based on visual inspection of waveforms, auto-correlograms, and cross-correlograms to obtain well-isolated single units. Firing rate across time for each cluster was also visually examined to ensure that the cell fired consistently across the recording session. Example waveforms from units that reached our criteria are shown in Supplementary Figure 1.

### 2.8 Mean firing rate

Mean firing rate (total number of spikes divided by total duration of maze session) was computed for each single unit for all no-tone and tone trials. Only instances in which the animal was moving 6+ cm/s were included. In addition, only cells which spiked 20+ times throughout the session were included. The proportional change in firing rate between no-tone and tone trials was calculated as (A-B)/(A+B). Therefore, a positive value implies an increase in firing rate from no-tone to tone trials. Mean firing rate was also examined for each zone of the maze and tone condition. Cells were only included if they had a combined 20+ spikes from both tone conditions with the given zone.

### 2.9 Classification of putative cell type

Identification of putative interneurons was based on single-units with spike width < 350 μs and firing rate > 7.5 Hz, and these were excluded from rate map analyses. Putative pyramidal (complex spike) cells were identified based on single-units having spike width > 350 μs. Only putative pyramidal cells with firing rate ≥ 0.1 Hz were included in the rate map analyses (Figure 4A).

The anatomical classification of hippocampal subregions was based on Jin and Lee (2021). However, because of clear changes in the spatial information content of place cells from AP −5.8 and on, in the current study the intermediate hippocampus was divided into anterior (aIH; AP −4 to AP −5.7) and posterior (pIH; AP −5.8 and AP −6.5) subregions. This then allowed for assessment of gradual changes in cell properties along the longitudinal axis.

### 2.10 Firing rate maps and place field characteristics

The environment was divided into 0.3 × 0.3 cm bins. The total number of spikes that occurred in each spatial location bin was summed and smoothed with a 10 × 10 bin Gaussian filter with a standard deviation of approximately 2.5 bins. The total amount of time that the rat spent in each spatial location bin was also summed and smoothed using the same parameters. Smoothed rate maps were calculated as the smoothed total number of spikes divided by the smoothed time spent in each spatial location bin (example firing rate maps in Figure 4D). Bins with insufficient sampling (< 1 ms in time spent) were regarded as unvisited and were not included in the rate map. Rate maps were only calculated for periods when the rat was moving at a robust speed (> 6 cm/s) to control for possible influence of stationary periods. Analysis of rate maps was separated into two directions to control for place cell directional selectivity (Markus et al., 1995). Place fields were defined as an area of at least 500 bins sharing adjacent edges, with a firing rate greater than 20% of the peak firing rate of the cell for the given experimental session. Percentage of active bins were calculated as the number of bins with a firing rate > 20% of the peak firing rate of the cell during the session. The percentage of active bins was also analyzed by zone and trial type for all complex spike cells in a similar manner, but instead using the peak firing rate for the given zone/trial type conditions.

### 2.11 Spatial correlations

Place field stability was calculated by measuring the correlation of the rate maps for the first and last five trials for the given experimental condition (i.e., direction × tone condition). A prerequisite for performing a rate map correlation was a minimum of one place field during a recording. Correlations between trial types were computed only if the cell fired at least 20 spikes in each trial type. The spatial information (bits per spike) for each neuron was calculated using the smoothed rate maps


$$\sum_{i}P_{i}\,\frac{R_{i}}{R}\,\log_{2}\,\frac{R_{i}}{R}$$

where *i* is the bin number, *P_i_* is the probability of occupancy in bin *i* (total time spent at bin *i* divided by total time spent through the maze session), *R_i_* is the mean firing rate for bin *i*, and *R* is the mean firing rate of the cell during the maze session (Skaggs et al., 1992).

As used in previous studies (Markus et al., 1995; Oler and Markus, 2000; Oler et al., 2008), relative rate map scores were calculated using the within- and between-trial-type rate map correlations as follows:


$$R_{\text{tone}}=\frac{\left(r\,\mathrm{within}-\mathrm{condition}\right)}{\left(r\,\mathrm{within}-\mathrm{condition}\right)+\left(r\,\mathrm{between}-\mathrm{conditions}\right)}$$

This adjusts for each cell’s reliability and gives a more accurate comparison of rate maps between tone conditions. As used in Oler et al. (2008), cells with a relative score of 0.55+ were categorized as either appeared, disappeared, or changed. Appeared was defined as a cell with a relative score of 0.55+ in which a place field was detected in the tone but not no-tone condition. Disappeared was defined as a cell with a relative score of 0.55+ in which a place field was detected in the no-tone but not the tone condition. Changed was defined as a cell with a relative score of 0.55+ which had a field in both tone conditions.

### 2.12 Histology

At the conclusion of the experiments, rats were euthanized with CO_2_, and intracardially perfused with phosphate buffered saline (PBS) followed by 4% paraformaldehyde in PBS. Brains were post-fixed for at least 24 h in 4% paraformaldehyde in PBS. Brains were sectioned 75 μm thick with a vibratome (VT1000S; Leica), mounted on glass microscope slides and covered with Prolong Gold Antifade mounting medium. Tetrode tracks were examined using a Keyence BZ-X810 fluorescence microscope to determine locations of recorded single-units.

### 2.13 Experimental design and statistical analyses

The design of this experiment was within-subjects, as cells were compared between no tone and tone conditions within experimental sessions. Comparison of cells across hippocampal subregions was treated as a between-subjects factor. Behavioral results were analyzed using paired *t*-tests to show changes in behavior between tone conditions. Mean firing rate was analyzed using repeated-measures ANOVAs, with tone as a within-subjects factor and subregions as a between-subjects factor. Any one-way comparison of subregions was analyzed using a one-way, between-subjects ANOVA. All post hoc comparisons were performed using a Bonferroni correction to account for multiple comparisons.

### 2.14 Code accessibility

All unit analysis was performed using custom MATLAB code. This code will be made available upon request.

## 3 Results

### 3.1 Behavioral changes in response to tone

To ensure animals were hesitating specifically to the tone and not to the shock itself, time spent in each zone in tone-shock and tone-only (no shock) control trials was compared. For each zone, we found no difference between the amount of time spent in tone-shock versus tone-only trials (paired *t*-tests: shock zone, t_5_ = 2.83, *p* = 0.065; near exit zone, t_5_ = −1.51, *p* = 0.095; all other zones, *p* > 0.1; Shapiro-Wilk tests for normality, all *p* > 0.05). Subsequently, the data for the tone-shock and tone-only trials were pooled for all subsequent analysis.

**FIGURE 2:**
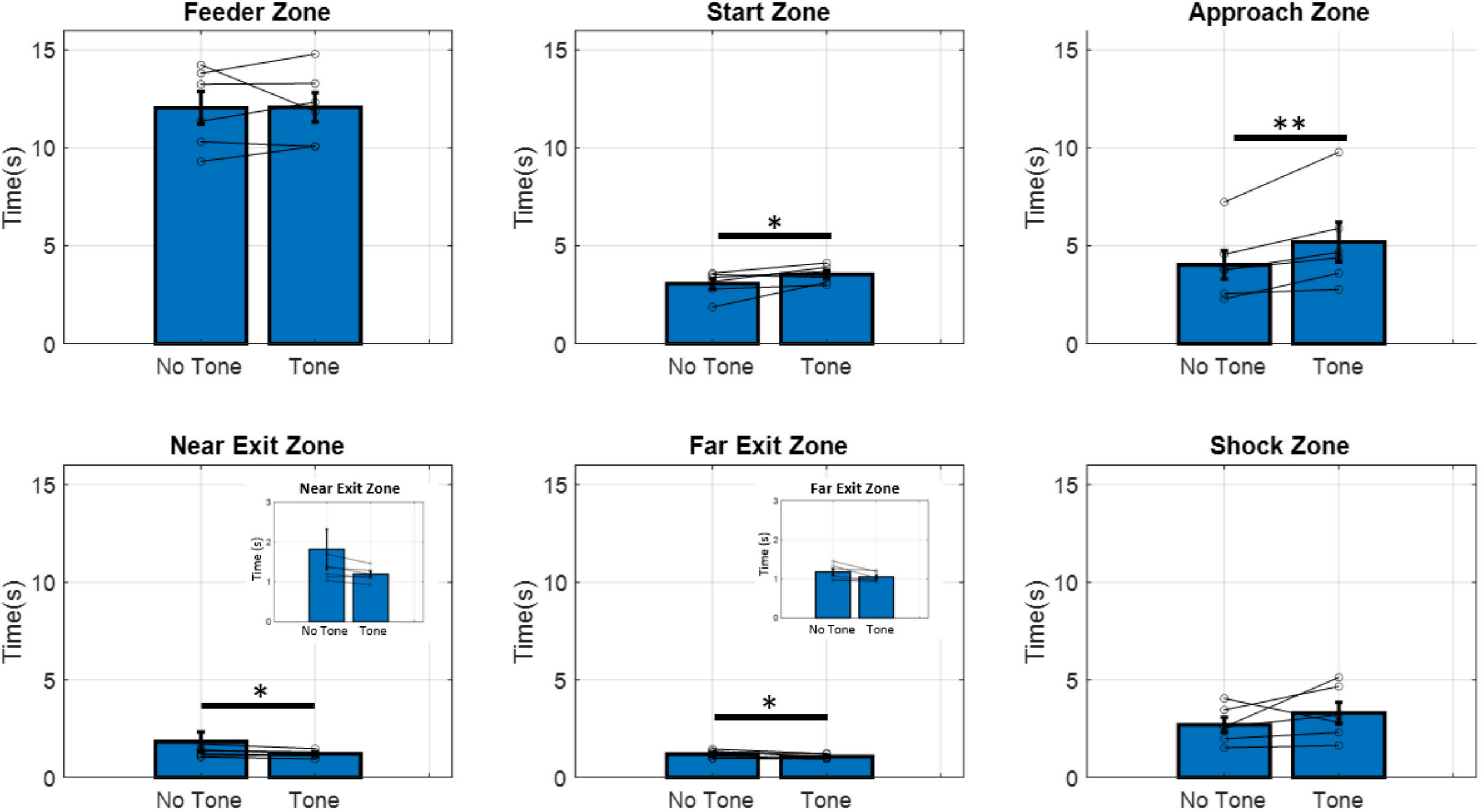
Time spent in each zone of the maze during no-tone and tone trials. Animals spent significantly more time in the start and approach zones during tone trials compared to no-tone trials. Animals spent significantly less time in the near and far exit zones during tone trials compared to no-tone trials. Inserts show the same data with a smaller scale. Paired *t*-tests significance levels are shown above bars. *p < 0.05, **p < 0.01. (*n* = 6).

To confirm that animals could discriminate between trials with the tone and trials without the tone, we measured the time spent in each zone on the maze (Figure 2) and compared time spent in the no-tone and tone conditions. No difference in time spent was seen in the feeder zone (paired t_5_ = −0.055, *p* = 0.48), while animals showed increased hesitation (latency) to the tone in the approach zone (t_5_ = −3.52, *p* < 0.01), and the start zone (t_5_ = −2.44, *p* < 0.05). This indicates animals associated the tone with the active shock zone, increasing hesitation in zones leading up to the shock zone. Interestingly, animals also showed a decrease in time spent on tone trials in the near exit zone (t_5_ = −3.03, *p* < 0.05) and far exit zone (t_5_ = −2.33, *p* < 0.05). In other words, animals “fled” from the shock zone faster after receiving the shock. Lastly, animals showed no difference in time spent in the shock zone in response to the tone (t_5_ = −1.179, *p* = 0.146). This null result may be due to the small size of the shock zone, making it difficult to detect any behavioral differences in this area.

### 3.2 Change in spatial information along the dorsal-intermediate axis

**FIGURE 3:**
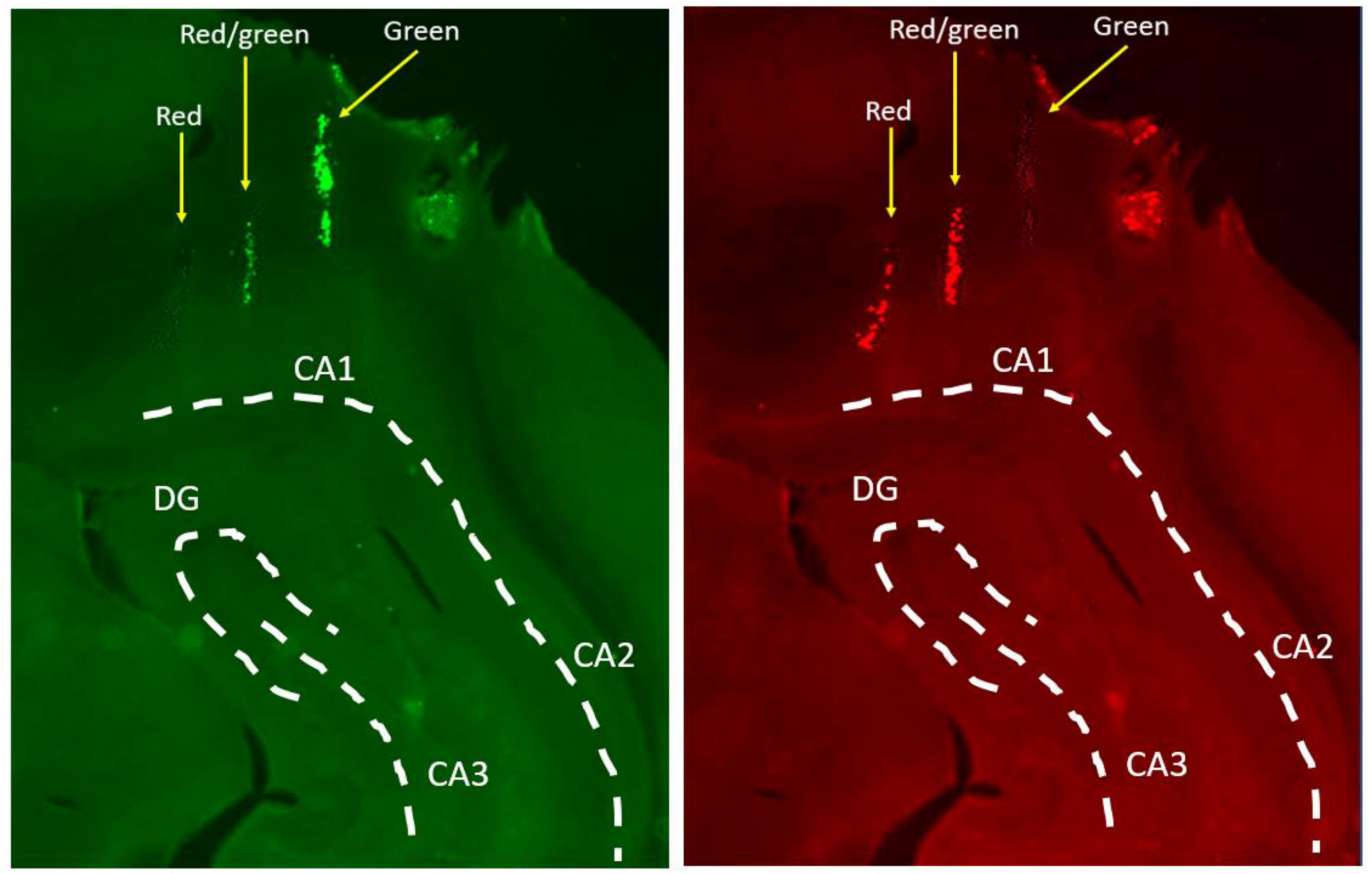
Confirmation of tetrode locations in the hippocampus. Tetrodes were marked with fluorescent microspheres which allow for more precise tracking of individual tetrodes in the brain. This example shows three tetrodes entering the aIH, one with red microspheres only, one with green microspheres only, and one with a combination of red and green. Tissue was imaged with separate filters for green (left) and red (right) fluorescence and then compared. White lines overlayed to illustrate anatomical boundaries (CA1, cornu Ammonis 1; CA2, cornua Ammonis 2; CA3, cornu Ammonis 3; DG, dentate gyrus).

**FIGURE 4:**
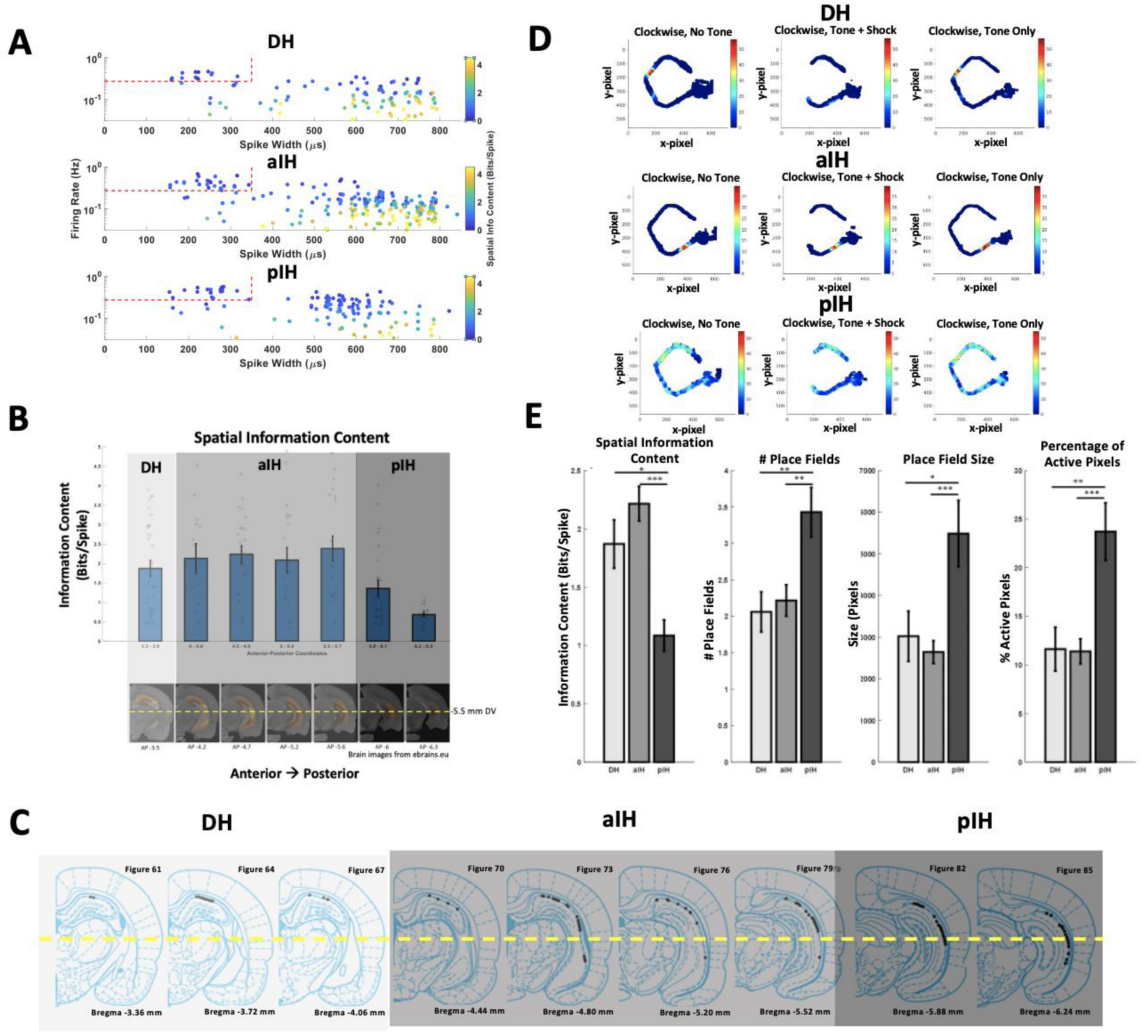
**(A)** Firing rate, spike width, and spatial information content for each cell in each hippocampal subregion. Red dashed lines mark the cutoff values for classifying interneurons. **(B)** Spatial information content of place cells along the dorsal-intermediate axis (top) and corresponding position in the brain for each bin (bottom). Spatial information showed a marked decrease in more posterior regions of the intermediate hippocampus. The number of cells is indicated at the bottom of each bar. The yellow line indicates the area in which cells were not included in analysis (ventral hippocampus). **(C)** Tetrode locations from the current study grouped into DH, aIH, and pIH based on change in spatial information content. **(D)** Example rate maps for a cell in the DH (top), aIH (middle), and pIH (bottom). These examples show rate maps for each tone condition in the clockwise direction on the maze. **(E)** General place cell characteristics in each subregion. Place cells showed distinct changes in spatial information content, number of place fields, place field size, and percentage of active pixels on the maze in the pIH compared to the DH and aIH. (DH: *n* = 31; aIH: *n* = 71; pIH: *n* = 34). Bonferroni-corrected *post hoc* comparisons shown above bars. *p* < 0.05*; *p* < 0.01**; *p* < 0.001***. Example brain images were gathered from ebrains.eu.

Tetrode recording locations throughout the CA1 cell layer were confirmed histologically using fluorescent microsphere labeled tracks (Figure 3) and/or, implant location and depth. Figure 4C shows confirmed tetrode locations in each hippocampal subregion as classified based on a change in spatial information.

Given the fact that our electrode array allowed for continuous sampling along the dorsal-intermediate axis, we were interested in determining the progression of change in place field characteristics as shown by Jung et al. (1994). Recordings were divided into the dorsal hippocampus (AP −3.3 to AP −3.9) and additional bins of 0.2–0.4 mm beyond the dorsal hippocampus (< AP −3.9). Using this split, we found a decrease in spatial information across the dorsal-intermediate CA1 axis (one-way independent measures ANOVA F_6,142_ = 4.97, *p* < 0.001) and a marked decrease in spatial information content from AP −5.7 to AP −5.8 (Bonferroni corrected paired comparisons, Figure 4B).

For all following analysis, the data were divided into the dorsal hippocampus (DH; AP −3.3 to AP −3.9), the anterior intermediate hippocampus (aIH; AP −4 to AP −5.7), and the posterior intermediate hippocampus (pIH; AP −5.8 to AP −6.5). Place field characteristics were analyzed across these three subregions (Figure 4E). As in the fine-grained comparison, spatial information content decreased across these three subregions (one-way independent measures ANOVA F_2,146_ = 12.81, *p* < 0.001), with a difference between the DH and pIH (Bonferroni-corrected *p* < 0.05), as well as the aIH and pIH (*p* < 0.001). There was an increase in the number of place fields across the three subregions (F_2,146_ = 6.69, *p* < 0.01) with significant differences between the DH and pIH (*p* < 0.01), as well as the aIH and pIH (*p* < 0.01). And there was also a significant increase in place field size across the three subregions (F_2,146_ = 8.87, *p* < 0.001), with significant differences between the DH and pIH (*p* < 0.05), as well as the aIH and pIH (*p* < 0.001). Lastly, we found an increasing percentage of active pixels across the three subregions (F_2,146_ = 11.2, *p* < 0.001) with a significant difference between the DH and pIH (*p* < 0.01), as well as the aIH and pIH (*p* < 0.001). Together, these results suggest that the DH and aIH show similar place field characteristics, while the pIH shows clear differences.

### 3.3 Firing rate change in response to tone and maze location

**FIGURE 5:**
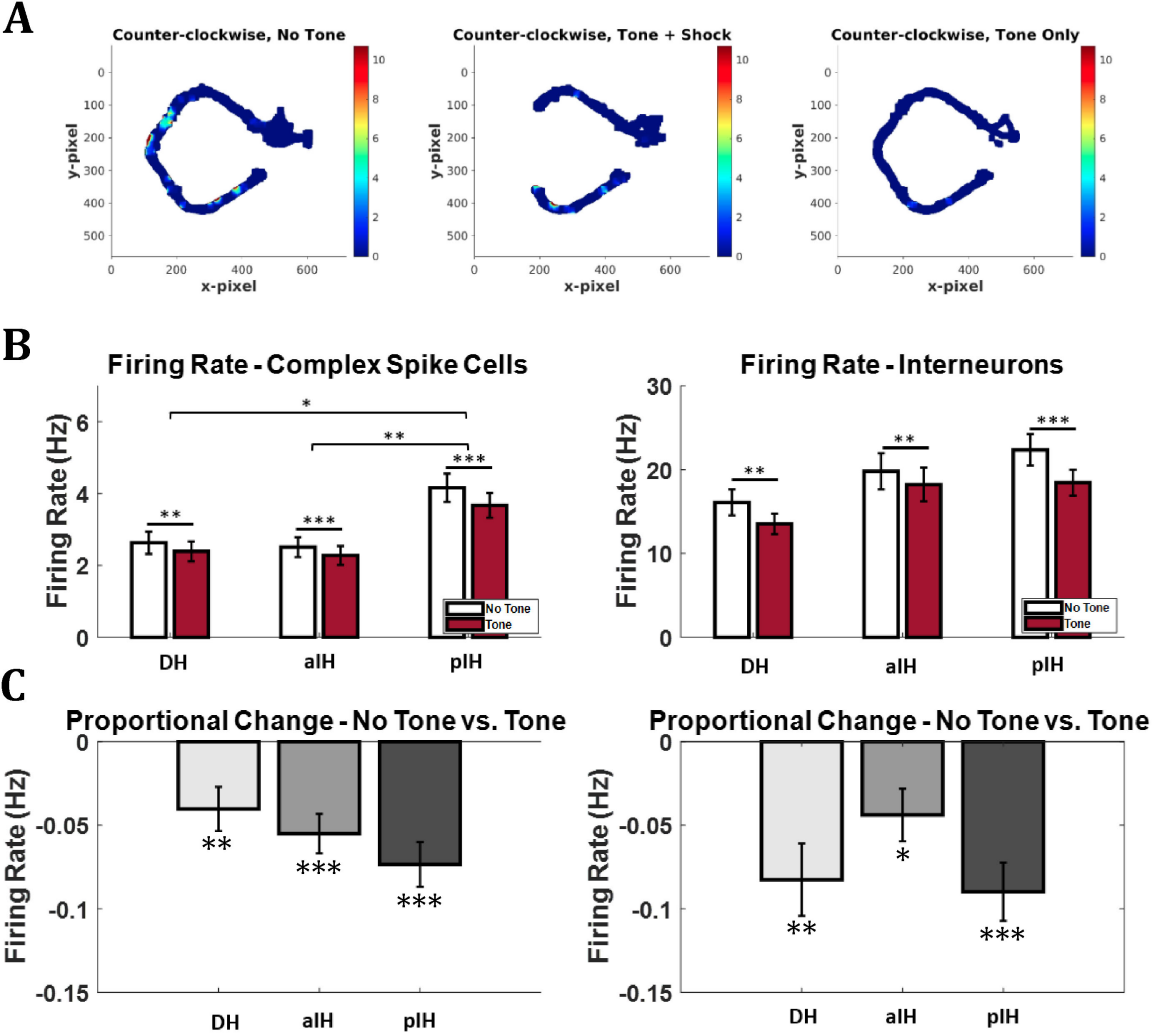
Firing rate change in response to tone. **(A)** Example firing rate map (DH). The cell showed a decrease in firing from the no tone (safe) to tone (unsafe) conditions. Note the lack of data near the shock zone in the Tone+Shock condition, the probe Tone only (no shock) trials provided the data for this part of the maze. Note also the disappearance of the place field in the approach zone of the maze. Bonferroni-corrected pairwise comparisons shown above bars. **(B)** Overall mean firing rate for entire recording session by brain area and tone condition. **(C)** Proportional change in firing between no tone and tone conditions in complex spike cells (left) and interneurons (right). One-sample *t*-tests versus zero are shown below bars. (Complex sike cells – DH: *n* = 91; aIH: *n* = 182; pIH: *n* = 109; Interneurons – DH: *n* = 13; aIH: *n* = 25; pIH: *n* = 19). *p* < 0.05*; *p* < 0.01**; *p* < 0.001***.

Next, we examined raw firing rate changes across hippocampal CA1 subregions and tone conditions. Figure 5 shows an example of a cell that reduced its firing during the tone trials. Figure 5B shows the general firing rate of complex spike cells and interneurons across the three subregions and tone conditions. For complex spike cells, a two-way repeated measures ANOVA revealed main effects for tone (F_1,379_ = 57.8, *p* < 0.001) and subregion (F_2,379_ = 7.04, *p* < 0.001) and an interaction between these two factors (F_2,379_ = 4.20, *p* < 0.05). Bonferroni-corrected pairwise comparisons also indicate a significant decrease in firing rate in response to the tone in all three subregions (all *p* < 0.01) and a significantly higher general firing rate in the pIH compared to both the DH (*p* < 0.05) and aIH (*p* < 0.01). For interneurons, there was a main effect of tone condition on firing rate (two-way repeated measures ANOVA F_1,54_ = 45.6, *p* < 0.001 and a significant interaction between these two factors (F_2,54_ = 3.46, *p* < 0.05), but no main effect of subregion (F_2,54_ = 1.79, *p* = 0.177). Bonferroni-corrected pairwise comparisons also indicate a significant decrease in firing rate in response to the tone in all three subregions (all *p* < 0.01).

To account for cells with differing baseline firing rates, we also calculated the mean proportional change in firing rate from the no tone to the tone condition. For complex spike cells, a one-way ANOVA showed no difference in proportional change in firing between subregions (F_2,379_ = 1.29, *p* = 0.276). However, one-sample *t*-tests versus zero (no change) showed a significant proportional decrease in firing on tone trials in all regions (DH: t_90_ = 3.05, *p* < 0.01, aIH: t_181_ = 4.64, *p* < 0.001, pIH: t_108_ = 5.46, *p* < 0.001). Similarly, no difference in proportional change in firing was seen in interneurons across subregions (F_2,54_ = 2.19, *p* = 0.122). However, one-sample *t*-tests versus zero showed a significant proportional decrease in firing on tone trials in all regions (DH: t_12_ = 3.82, *p* < 0.01, aIH: t_24_ = 2.79, *p* < 0.05, pIH: t_18_ = 5.14, *p* < 0.001) (Figure 5C).

These results suggest that, compared to DH and aIH cells, complex spike cells in the pIH were more active overall throughout the behavioral task regardless of tone condition. In addition, these data show that both complex spike cells and interneurons across all subregions decrease firing during tone trials compared to no tone trials.

**FIGURE 6:**
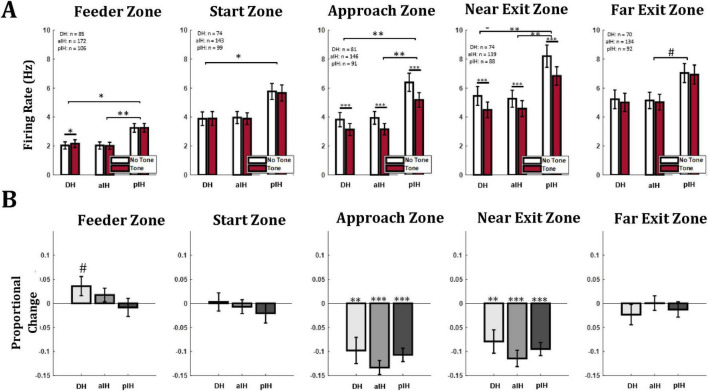
**(A)** Mean firing rate of all cells in each hippocampal subregion and each maze zone. **(B)** Proportional change in firing rate between no tone and tone trials. Bonferroni-corrected pairwise comparisons between subregions are shown in brackets above bars. Similar comparisons between no tone and tone trials within subregion are shown directly above bars. *p* < 0.05*; *p* < 0.01**; *p* < 0.001***. The symbol “#” indicates a *p* value between 0.05 and 0.1.

Next, we examined firing rate in each zone of the maze. In the feeder zone, there was a main effect for subregions (F_2,360_ = 6.08, *p* < 0.01) but there was no effect of tone (repeated measures ANOVA, F_1,360_ = 1.60, *p* = 0.21), and no interaction (F_2,360_ = 1.79, *p* = 0.17). Pairwise comparisons with a Bonferroni correction indicate a higher overall firing rate in the pIH compared to the DH (*p* < 0.05) and aIH (*p* < 0.01), as well as a higher firing rate on tone trials compared to no tone trials in the DH (Figure 6A). Here the proportional change in firing between no tone and tone trials was not substantially different between subregions (one-way ANOVA F_2,360_ = 1.37, *p* = 0.26) and there were not consistent changes in firing between tone and no-tone trials (one-sample *t*-tests versus zero: DH (t_84_ = 3.82, *p* = 0.074); aIH (t_171_ = 0.22, *p* = 0.22); pIH (t_105_ = −0.45, *p* = 0.65), (Figure 6B).

Similarly, in the start zone, there was a main effect of subregion (F_2,313_ = 4.59, *p* < 0.05), but no differences due to tone (repeated-measures ANOVA, F_1,313_ = 0.53, *p* = 0.47), and the interaction (F_2,313_ = 0.20, *p* = 0.82) were not statistically significant. Pairwise comparisons with a Bonferroni correction again indicate a higher overall firing rate in the pIH compared to the DH (*p* < 0.05) and aIH (*p* < 0.05) (Figure 6A). As in the feeder zone, the proportional change in firing between no tone and tone trials did not substantially differ between subregions (one-way ANOVA, F_2,313_ = 0.38, *p* = 0.69), and there was not a consistent proportional change in firing between tone and no-tone trials (one-sample *t*-tests versus zero: DH (t_73_ = 0.15, *p* = 0.88); aIH (t_142_ = −0.48, p = 0.63); pIH (t_98_ = −1.01, *p* = 0.32), (Figure 6B).

In the approach zone, there were main effects of tone (repeated measures ANOVA, F_1,315_ = 118.3, *p* < 0.001) and subregion (F_2,315_ = 7.09, *p* < 0.001), and an interaction between these two factors (F_2,315_ = 3.50, *p* < 0.05). Pairwise comparisons with a Bonferroni correction again indicate a higher overall firing rate in the pIH compared to the DH (*p* < 0.05) and aIH (*p* < 0.001), as well as a decreased firing rate during tone trials compared to no tone trials in the DH (*p* < 0.001), aIH (*p* < 0.001), and pIH (*p* < 0.001) (Figure 6A). There were not substantial differences between subregions in the proportional change in firing between no tone and tone trials (one-way ANOVA F_2,315_ = 1.14, *p* = 0.32). However, for proportional change in firing, one-sample *t*-tests versus zero (no change) showed a significant decrease on tone trials in the DH (t_79_ = −3.54, *p* < 0.01), aIH (t_145_ = −9.21, *p* < 0.001), and pIH (t_90_ = −7.75, *p* < 0.001) (Figure 6B).

We also found differences in firing as animals left the shock zone in the near exit zone. Here, there were also main effects for tone (repeated measures ANOVA, F_1,298_ = 79.3, *p* < 0.001) and subregion (F_2,298_ = 5.27, *p* < 0.01) and an interaction between these two factors (F_2,298_ = 3.59, *p* < 0.05). Pairwise comparisons indicate a higher overall firing rate in the pIH compared to the DH (*p* < 0.05, Bonferroni corrected) and aIH (*p* < 0.001), as well as a decreased firing rate during tone trials compared to no tone trials in the DH (*p* < 0.001), aIH (*p* < 0.001), and pIH (*p* < 0.001) (Figure 6A). Again, there were not substantial differences in the proportional change in firing between no tone and tone trials across subregions (one-way ANOVA, F_2,298_ = 0.93, *p* = 0.39), but there were differences versus zero for each region on tone trials [one-sample *t*-test DH (t_73_ = −3.26, *p* < 0.01), aIH (t_138_ = −6.75, *p* < 0.001), and pIH (t_87_ = −7.10, *p* < 0.001)] (Figure 6B).

Lastly, as animals traversed the far exit zone, we found that there was not a main effect of tone (repeated measures ANOVA F_1,293_ = 2.24, *p* = 0.14), and no interaction between these two factors (F_2,293_ = 0.11, *p* = 0.90), but there was a significant main effect of subregion (F_2,293_ = 3.05, *p* < 0.05). Again, there were not substantial differences in the proportional change in firing between no tone and tone trials across subregions (one-way ANOVA, F_2,293_ = 0.48, *p* = 0.62) (Figure 6B). And here, there was no significant proportional change in firing between tone and no-tone trials (one-sample *t*-tests versus zero: DH (t_69_ = −1.10, *p* = 0.28); aIH (t_133_ = 0.03, *p* = 0.98); pIH (t_88_ = −0.86, *p* = 0.39), (Figure 6B).

Taken together, these data indicate that while overall there was a higher firing rate in the pIH, all three regions showed a decrease in firing rate during tone trials specifically in the maze zones adjacent to the shock region.

### 3.4 Differences in remapping across subregions in relation to maze location

In order to compare how the spatial representation of the CA1 cells responded to change in emotional context, we used three different analyses: rate map correlations, summaries of place field changes (appear/disappear/moved), and an analysis of regions of active cell firing.

#### 3.4.1 Rate map correlations

**FIGURE 7:**
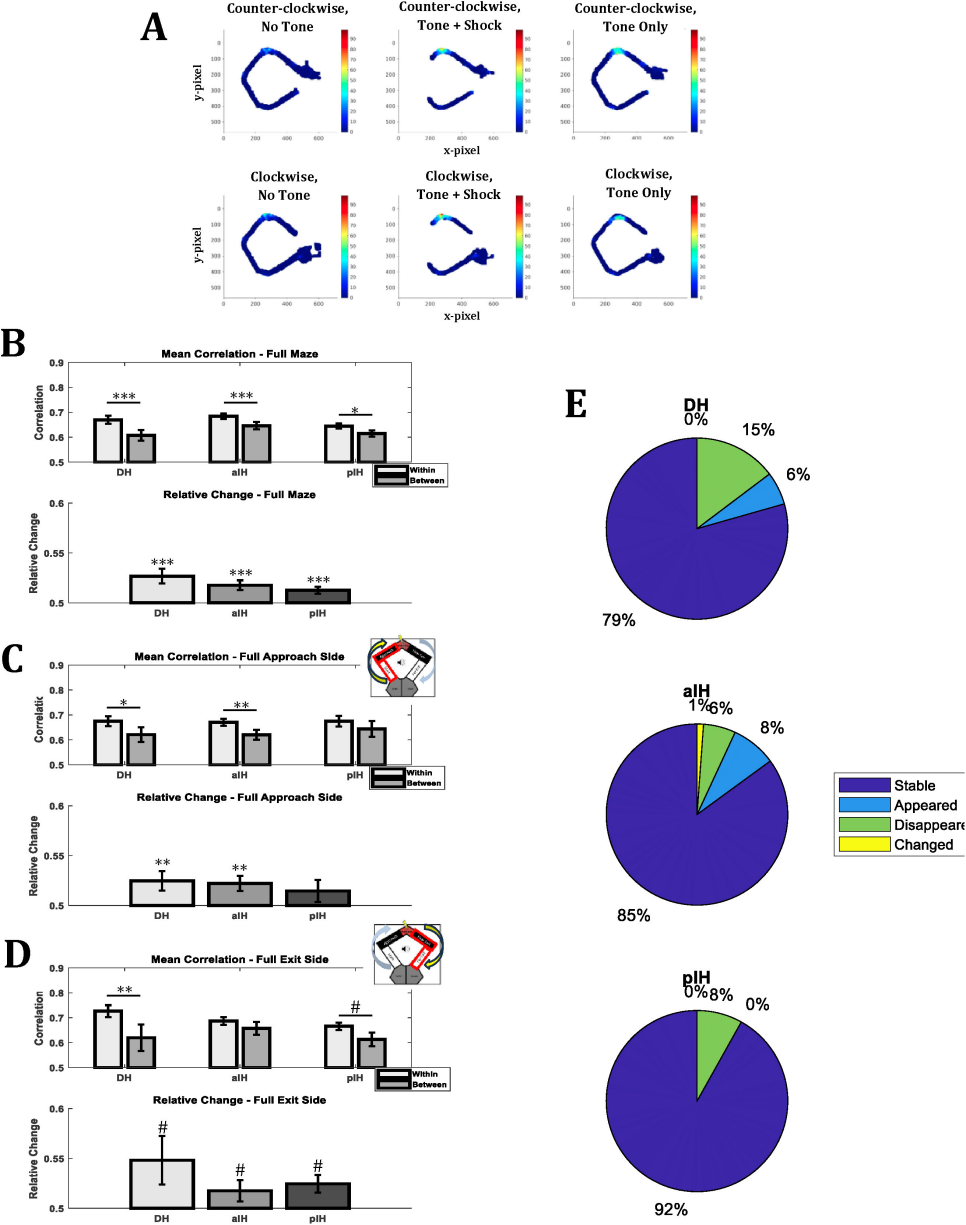
Mean rate map correlations of place cells for the same tone condition (within; i.e., no tone - no tone and tone - tone) or different tone conditions (between; no tone - tone and tone - no tone) within experimental sessions. **(A)** Example of rate remapping in a DH place cell. **(B)** Comparison of rate maps for the entirety of the maze. **(C)** Comparison of rate maps for the approach side of the maze (i.e., Approach zone and start zone, indicated by insert). **(D)** Comparison of rate maps for the exit side of the maze (i.e., Near exit zone and far exit zone, indicated by insert). **(E)** Percentage cells that were stable, appeared, disappeared, or changed from the no tone to the tone condition. Bonferroni-corrected pairwise comparisons are shown above bars. *P* < 0.05*; *p* < 0.01**; *p* < 0.001***; 0.1 > *p* > 0.05#. (Full maze – DH: *n* = 34; aIH: *n* = 87; pIH: *n* = 37) (Approach side – DH: *n* = 20; aIH: *n* = 40; pIH: *n* = 14) (Exit side – DH: *n* = 11; aIH: *n* = 37; pIH: *n* = 20).

To examine differences in how space was coded based on maze location, we compared rate maps between no tone and tone trials for the full maze, and we also separated rate map correlations for the approach side of the maze (i.e., the zones leading up to the shock zone) and the exit side of the maze (i.e., the zones following the shock zone). Rate map correlations were calculated for the first and second half of trials during experimental recordings. Importantly, this was done both within tone conditions and between tone conditions to provide a baseline for place field stability in each subregion. Relative rate map scores were also calculated in order to show the relative difference of within versus between trial type correlations. An example of a remapping cell is shown in Figure 7A.

For rate map comparisons of the entire maze (Figure 7B), we found a main effect of tone condition (two-way ANOVA F_1,155_ = 29.1, *p* < 0.001), but not subregion (F_2,155_ = 2.11, *p* = 0.13), and no interaction between these factors (F_2,155_ = 1.18, *p* = 0.31). Bonferroni-corrected pairwise comparisons also indicate a significant reduction in correlation when comparing correlations within a tone condition to between tone conditions in the DH (*p* < 0.001), the aIH (*p* < 0.001), and the pIH (*p* < 0.05). In addition, we examined the relative change in correlation of the within vs. the between conditions (see section “2 Materials and methods”). In all subregions, there was a greater change in degree of correlation between conditions than within conditions (one-sample *t*-tests versus 0.5, DH: t_33_ = 3.62, *p* < 0.001; aIH: t_86_ = 3.56, *p* < 0.001; pIH: t_36_ = 3.69, *p* < 0.001).

When comparing rate maps for only the approach side of the maze (Figure 7C) or only the exit side of the maze (Figure 7D), we also found that there was a main effect of tone condition (two-way ANOVA F_1,71_ = 11.95, *p* < 0.001 for approach, F_1,65_ = 13.88, *p* < 0.001 for exit), but no significant main effect of subregion (F_2,71_ = 0.11, *p* = 0.90 for approach, F_2,65_ = 0.67, *p* = 0.52 for exit) or interaction (F_2,71_ = 0.22, *p* = 0.8 for approach, F_2,65_ = 1.65, *p* = 0.2 for exit).

#### 3.4.2 Place field comparisons

We also evaluated the percentage of cells that were stable, appeared, disappeared, or changed for each subregion (Figure 7E). Similar to the correlation data, we found that there was more remapping (i.e., fewer cells were stable) across the no-tone to tone condition in the DH (21%) than in the aIH (15%) and pIH (8%).

#### 3.4.3 Regions of active cell firing

**FIGURE 8:**
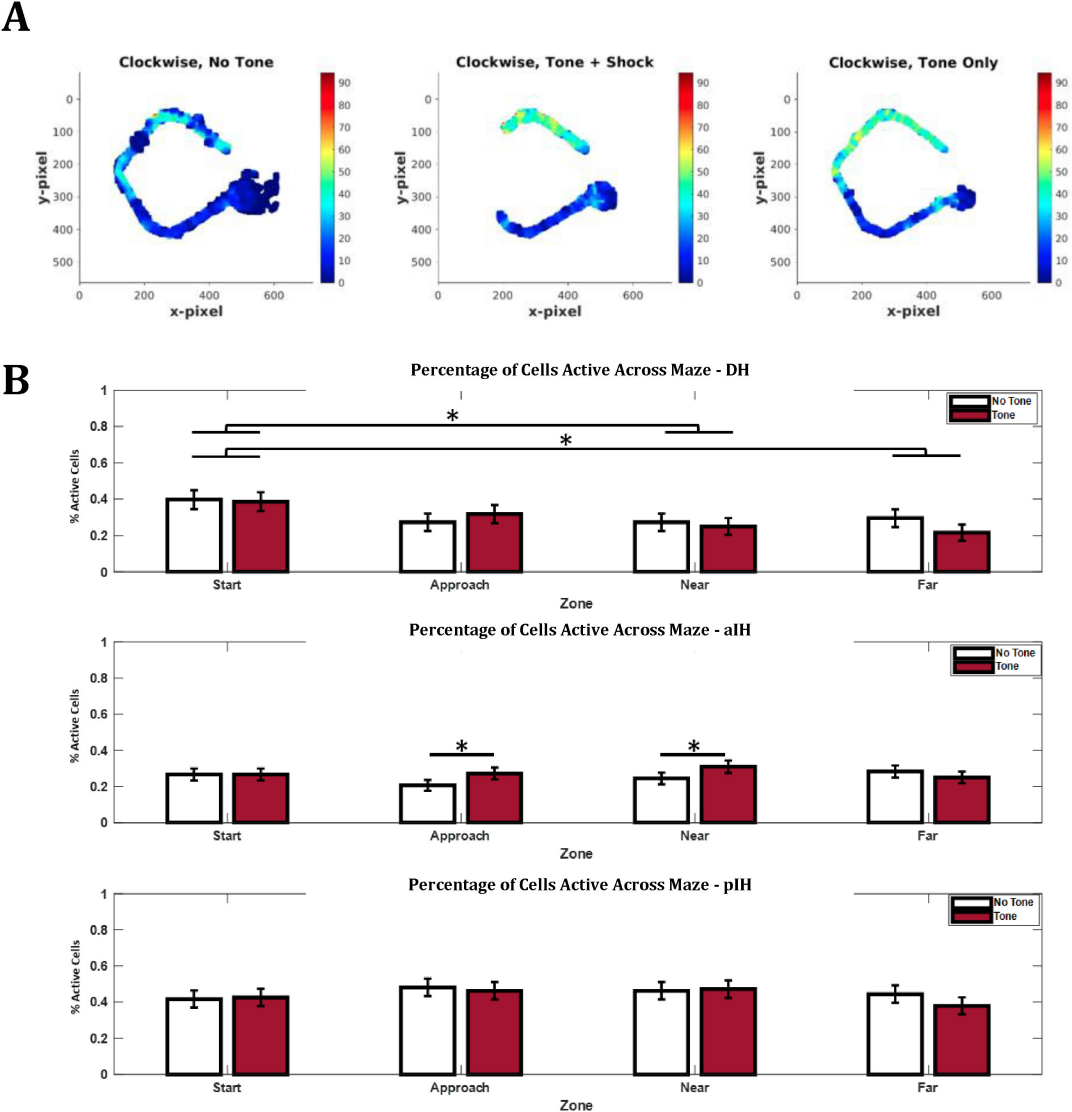
Differences in spatial representation of cells along dorsal-intermediate axis. **(A)** Example rate remapping illustrating the differences seen in the aIH. **(B)** Percentage of cells active in each tone/zone condition, maze zone, and subregion. Bonferroni-corrected pairwise comparisons are shown above bars. Active cells were defined as 25% of the zone having a firing rate > 20% of the peak firing for the given cell. The DH showed greater spatial selectivity for the start zone compared to the near and far exit zones. In addition, the aIH shows greater spatial selectivity for the approach and near exit zones during tone trials compared to no tone trials. The pIH shows no difference in spatial selectivity by zone or tone condition. (DH: *n* = 88; aIH: *n* = 184; pIH: *n* = 108). Pairwise comparisons with the Bonferroni correction are shown above bars. *p* < 0.05*.

Given the fact that CA1 cells in the pIH show a more dispersed spatial firing than the more dorsal regions (Figure 4E), we also examined the degree to which individual complex spike cells showed active firing in different parts (zones) of the maze. Here we defined active spatial firing as zones with firing > 20% above the peak firing rate of the cell (see section “2 Materials and methods”). For a cell to be considered “active” in a given zone, 25% or more of the zone must reach this firing criteria. This provides an index, at the population level, as to whether the DH, aIH, and pIH subregions preferentially represent certain parts of the maze and/or the presence of the tone (Figure 8A).

In the DH, we found that there was no main effect for tone on the percentage of active cells (two-way ANOVA, F_1,87_ = 0.44, *p* = 0.51), but there was a significant main effect of zone (F_3,261_ = 3.88, *p* < 0.05). There was no interaction between these two factors (F_3,261_ = 1.24, *p* = 0.30). Bonferroni-corrected pairwise comparisons indicated differences between the start and near exit zones (*p* < 0.05) and the start and far exit zones (*p* < 0.05). This may suggest a preference in the DH for representing the start zone of the maze (Figure 8B).

In the aIH, there were no main effects (F_1,183_ = 3.03, *p* = 0.08 for tone, F_3,549_ = 0.56, *p* = 0.64 for zone) or interaction (F_3,549_ = 2.29, *p* = 0.08). However, Bonferroni-corrected pairwise comparisons indicated differences between no tone and tone trials in the approach zone (*p* < 0.05) and the near exit zone (*p* < 0.05). These data may suggest that the aIH shows increased representation of areas closer to the shock zone specifically during tone trials (Figure 8B).

Similarly, in the pIH, there were no main effects (F_1,107_ = 0.62, *p* = 0.43 for tone, F_3,321_ = 1.21, *p* = 0.31 for zone) or no interaction (F_3,321_ = 0.70, *p* = 0.56). These results suggest that the pIH does not show substantial changes in spatial selectivity of the overall population for specific zones of the maze during either trial type (Figure 8B).

Taken together, these data suggest that remapping between tone conditions is seen across all subregions to some extent (Figure 8A). Contrary to our hypothesis, remapping was stronger in the DH compared to the aIH and pIH, with remapping occurring both on the approach and exit sides of the maze. These data also suggest that place cells in the aIH are particularly responsive to change in the approach side of the maze and not the exit side of the maze.

### 3.5 Anticipation of tone information in DH and aIH, but not pIH

**FIGURE 9:**
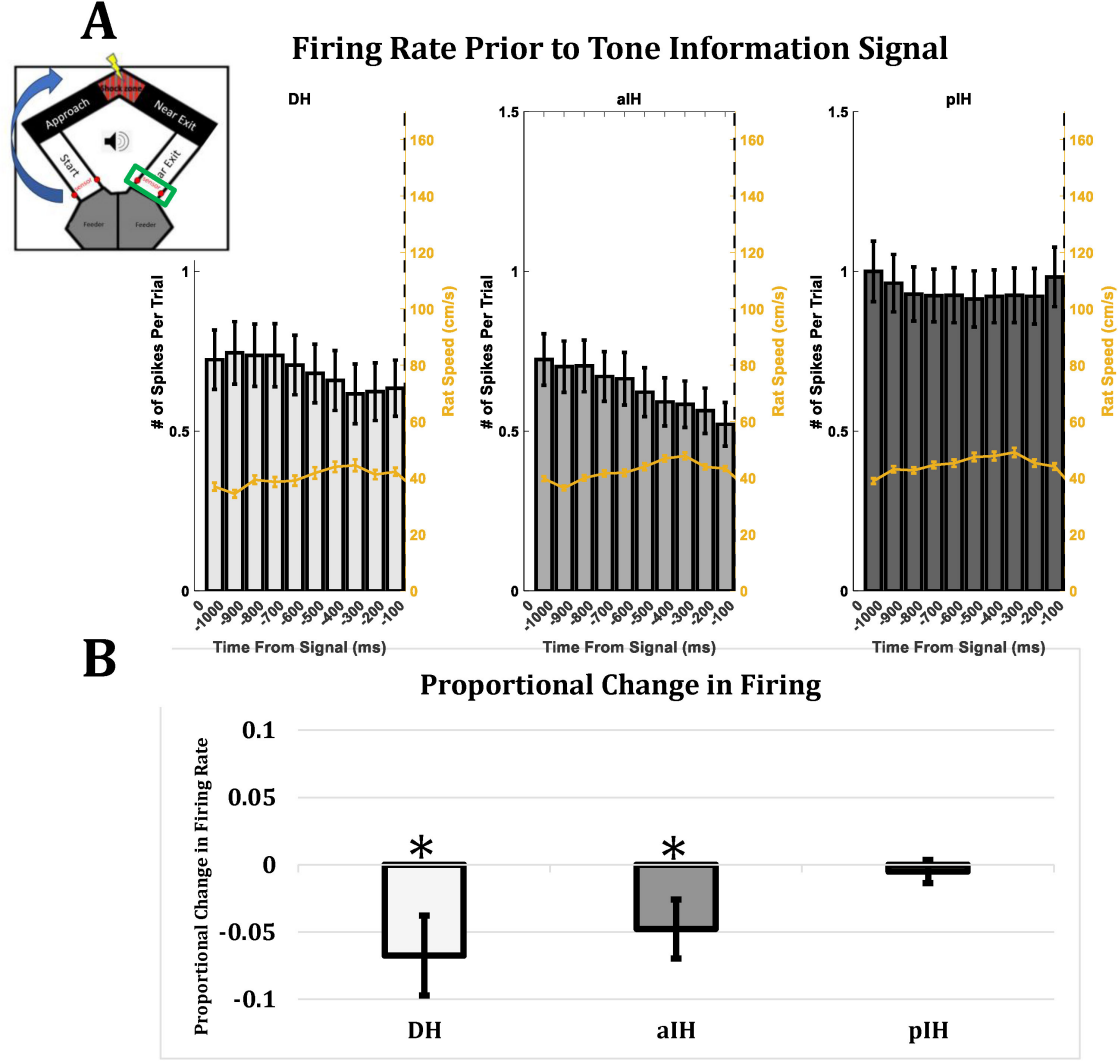
**(A)** Firing rate in each subregion leading up to a possible change in tone information. Yellow line indicates animal speed. Insert in top left corner indicates the part of the maze (green rectangle) where the rat crosses the sensors and triggers a change (or no change) in tone. **(B)** Proportional change in firing rate from bins –600 to –300 compared to bins –300 to 0. A decrease in firing rate leading up to a change in tone information was seen in DH and aIH, but not pIH. This change was seen even while animal speed stayed consistent (DH: *n* = 57; aIH: *n* = 117; pIH: *n* = 79). One-sample *t*-tests compared to zero are shown above bars. *P* < 0.05*.

Another way of analyzing hippocampal processing of emotional information is to examine cell activity during the time interval leading up to the upcoming trial, as animals came to the end of each trial and approached the sensors (see Figure 1) which trigger the conditions of the next trial (a possible change in tone: onset, offset, or no change). Therefore, we analyzed firing rate during the one-second window leading up to the animal crossing the sensor in the far exit zone at the end of each trial. Figure 9A shows the mean firing rate and animal speed (yellow line) in each subregion in 100 ms bins leading up to the possible change in tone information. Figure 9B shows the proportional change in firing rate in each subregion from bins −600 to −300 compared to bins −300 to 0. Here, we found a decrease in firing in the DH (one-sample *t*-test compared to zero, t_54_ = −2.28, *p* < 0.05) and the aIH (t_115_ = −2.17, *p* < 0.05), but not the pIH (t_78_ = −0.59, *p* = 0.28) leading up to the tone information regarding the next trial.

These results suggest possible anticipation of tone information in the DH and aIH, but not the pIH. Taken together, our results generally indicate that these subregions have greater involvement in processing emotional information during this task.

## 4 Discussion

The current study examined single-unit activity along the CA1 dorsal-intermediate axis of the hippocampus as animals navigated in a changing emotional context. Consistent with previous findings, we found that spatial information content was lower in the pIH, as compared to the DH and aIH, but, in addition to studying spatial representation, the task here allowed hippocampal unit representation to be determined as emotional/motivational behavioral state varied between “safe” and “unsafe” situation. Crucially, the spatial configuration of the maze and the trajectory of the animal remained stable and only the emotional valence was manipulated.

Animals modified their behavior in response to the tone, suggesting that they are able to discriminate between safe and unsafe trials. No behavioral differences were seen between tone-shock trials and tone-only trials, indicating that the rats were responding to the tone, rather than somehow sensing the shock. Firing rates decreased in response to the tone in the regions adjacent to the shock zone in all hippocampal subregions, with less remapping to the tone along the dorsal-intermediate axis.

### 4.1 Difference between aIH and pIH in place field characteristics

In general, spatial firing in the DH and aIH was similar, while the pIH cells had less specific firing similar to what has been previously reported in ventral regions (Jung et al., 1994; Kjelstrup et al., 2008). This differentiation between unit activity in the aIH and pIH parallels previous findings using other approaches. Gene expression in the CA1 cell layer shows three distinct domains which delineate the dorsal, intermediate, and ventral hippocampus (Dong et al., 2009). However, CA3 can be divided into as many as nine domains (Thompson et al., 2008). This genetic heterogeneity, combined with the gradient of connectivity to other brain areas and changes in the intrinsic connectivity of the hippocampus described above, could give rise to many distinct functional domains along the dorsoventral axis.

An important point to consider with respect to the current results is the abrupt change in the intrinsic connectivity of the dorsal two-thirds of the hippocampus versus the ventral one-third. Longitudinal fibers within the CA3 and dentate gyrus do not appear to cross between these two regions, perhaps indicating some separation in the way these subregions function (Swanson et al., 1978; Amaral and Witter, 1989; Li et al., 1994; Ishizuka et al., 1990). In the current study, the DH and aIH approximately fall within the dorsal two-thirds of the longitudinal axis, and the pIH approximately falls within the ventral one-third.

Our results also indicate a higher firing rate in the pIH compared to the DH and aIH, regardless of the presence of the tone. If the pIH more closely resembles what is commonly considered the ventral hippocampus, this increased firing rate would be consistent with previous work showing greater involvement of the ventral hippocampus in anxiety-related behavior (Fanselow and Dong, 2010; Jimenez et al., 2018). Although there was no difference seen between tone conditions, it may be that regardless of tone presence, the maze area was generally anxiogenic after extensive learning.

### 4.2 Rate remapping

A general decrease in firing rate was seen across all subregions during tone trials compared to no tone trials, both in complex spike cells and interneurons. Furthermore, this decrease was limited to the zones adjacent to the shock zone. The reduction in firing rate during the tone trials may partially be due to changes in speed. However, rates decreased for *both* the approach and near exit zones when the tone was present despite the fact that animals spent *more time* in the approach zone during tone trials and *less time* in near exit zone. The decrease in firing rate may reflect a change in hippocampal firing patterns during (and immediately following) the animal’s decision to cross the shock zone. Our findings are in line with previous work suggesting non-spatial information may be coded by changes in firing rate (Allen et al., 2012; Leutgeb et al., 2005; Sanders et al., 2019; Lu et al., 2015).

While a reduction in pyramidal cell firing rate is often attributed to increased inhibition from interneurons, a decrease in firing was also seen in interneurons in the current study. Therefore, inhibition of putative pyramidal cells in this task may be mediated through another inhibitory mechanism. Importantly, this decrease in firing was seen equally in all three subregions (Figure 6B), indicating that in terms of raw average firing rate, these subregions react similarly, to the presence of the tone while close to the shock zone.

Also noteworthy is the finding that although behavioral differences were found in the start and far exit zones between tone conditions, no difference was seen in these areas in terms of firing rate. Therefore, changes in the animal’s behavior did not always align with the hippocampal activity. A possible explanation is that a change in firing in these areas was not detected because the change in behavior was more subtle in areas further from the shock zone.

The behavioral paradigm in the current experiment can also be thought of as an approach-avoidance conflict task. While the shock zone represents an aversive experience, crossing the shock zone is also associated with retrieval of a food rewards. Previous work has explored the contribution of the hippocampal subfields to this behavior. Schumacher et al. (2018) tested animals in an approach-avoidance conflict task in which they were presented with a maze arm which contained contextual cues associated with both positive (food rewards) and negative (foot shock) conditioned stimuli. Ventral CA3 inactivation increased approach behavior to a context with conflicting motivational cues, while ventral CA1 inactivation increased avoidance. Inactivation of the DH did not affect behavior in the task. Yeates et al. (2022) extended these findings to distinct pathways from the VH to the lateral septum. In the current study, when approaching and exiting the shock zone, a decrease in firing rate was found across both dorsal and intermediate CA1 cells.

Since the intermediate/ventral subregions of the hippocampus provide the primary hippocampal communication with motor and executive control regions such as the prefrontal cortex, amygdala, nucleus accumbens, septum, and hypothalamus (Bast, 2007; Bast et al., 2009; Sánchez-Bellot et al., 2022), one possibility is that the hippocampus initially is inducing a state of behavioral inhibition during conflict evaluation and the subsequent decrease in firing occurs during action (i.e., behavioral disinhibition) (Jarrard, 1973; Chan et al., 2001). Our findings are consistent with this idea that firing rate decreases could be related to an action/motivational response as animals transition from a state of conflict evaluation to a “go” state.

In this same vein, recent work by Patterson et al. (2025) shows that ventral CA1 input to the nucleus accumbens is involved in approach-avoidance decision making, as inhibition of this pathway resulted in increased decision-making time. Perhaps a similar mechanism is at play in the current study and therefore, ventral hippocampal activity in this task should be studied in future experiments.

### 4.3 Global remapping

Our results also suggest that global remapping occurred across trial types in all subregions, with the greatest effect in the DH. Given functional and anatomical differences along the dorsoventral axis of the hippocampus (Fanselow and Dong, 2010; Strange et al., 2014), this finding was unexpected. However, animals underwent extensive training on the task before recordings took place. This may be related to differential involvement, of the dorsal intermediate, and ventral hippocampus across learning (Ruediger et al., 2011; Bast et al., 2009). Jin and Lee (2021) used a similar experimental design to ours, but instead looked at how changes in rewards value affect place cell activity. They recorded cells during the first exposure to rewards value change, and found greater remapping in the IH compared to DH, as well as immediate changes in firing patterns of the cells in the IH but not the DH in response to a changing rewards value. However, Komorowski et al. (2013) showed that, in an odor contextual task, spatial representations in the VH evolve over a longer time frame compared to the DH. It is, thus, possible that there may be distinct patterns of remapping early versus late in learning for DH vs. VH. A recent study by Ormond et al. (2023) recorded dorsal place cells before and after an aversive shock in a task where trajectory was kept consistent. They showed that dorsal cells do partially remap in response to the shock and, interestingly, these specific cells are preferentially recruited into sharp-wave ripples. This therefore suggests that ripple events may be a mechanism for separating updated spatial maps from existing ones. Future work may examine differences in ripple events along the hippocampal long axis as animals acquire a task.

When classifying place fields as either appearing, disappearing, or changing location, we found less change in the pIH. Given the fact that almost no place fields changed location, it could indicate there is no global remapping and the source of change was rate remapping. However notably, there were no regional differences in the firing rate decrease across the three regions. Furthermore, the issue of remapping was also examined using a rate-map correlation analysis, which is less affected by overall changes in firing rate. The correlation analysis indicated that in addition to the overall rate changes there also seems to be reduced global remapping in the pIH.

Examining spatial firing patterns across all complex spike cells (i.e., not only place cells), we observed some differences in how units in the three hippocampal subregions coded for specific parts of the maze. In the DH, there was a greater percentage of cells active at the start of the maze compared to the end. Interestingly, the aIH had an increased percentage of cells active in the approach and near exit zones specifically when the tone was on. In contrast, the percentage of active cells in the pIH did not substantially vary with zone or tone condition. This was the only variable that showed a differentiation between the dorsal and aIH unit activity. While raw firing rates (Figure 6) indicate a decrease in the average firing rate in the approach and near exit zones in response to the tone, these data indicate a small subset of cells in the aIH that fire preferentially in these areas. This may reflect differences in connectivity of the aIH from the DH and pIH with the amygdala. Based on the pattern of connectivity between the amygdala and ventral two-thirds of the hippocampus, the aIH in the current study may be more strongly connected to the basolateral nuclei which process conditioned fear and auditory information (Pikkarainen et al., 1999; Fanselow and LeDoux, 1999; Petrovich et al., 2001; Kishi et al., 2006). This finding may suggest a unique role for the aIH.

One possibility is that because of the extensive training involved, perhaps the zones closer to the shock zone are aversive regardless of tone presence. However, when looking at the percentage of active cells across the maze, there were no main effects of zone in any subregion. This suggests that even though the behavioral data indicate an aversion to the tone and zones close to the shock zone, the aIH seems to be the only subregion in which this effect is reflected physiologically.

One limitation of the current study regarding remapping analysis is that, because the pIH showed lower spatial information than the other subregions, this may inherently make global remapping (Figure 7E) less detectable. However, comparing rate map correlations both within and between-tone conditions (Figures 7B–D) accounts for this by including the within-tone-condition as a baseline for each subregion. Therefore, while global remapping may be harder to detect with lower spatial information in the pIH, rate remapping is still fairly quantified.

Although there was not sufficient data in the current study, another interesting analysis would be to examine spatial firing patterns of *only* place cells, with respect to maze location, tone presence, and long axis position. Future studies should assess this to help clarify how place cell function may differ depending on long axis position.

### 4.4 Tone information

As animals came to the end of each trial and approached the sensors which trigger the conditions of the next trial (a possible change in tone: onset, offset, or no change), cell firing in the DH and aIH was found to decrease, while the pIH remained stable. This may indicate a role for the DH and aIH in the anticipation of possible tone information. The role of the hippocampus in processing time has been heavily studied (Banquet et al., 2021). However, less work has examined how this processing might differ along the dorsoventral axis of the hippocampus. The differences observed here could also reflect a change along the long axis in processing or anticipating conditioned stimuli, specifically. This may be consistent with previous work showing that more ventral regions of the hippocampus are linked to unconditioned fear and anxiety (Jimenez et al., 2018) and have greater connectivity to hypothalamus (Risold and Swanson, 1996).

Another possibility is that these differences are related to a rewards expectation as the animal approaches the feeder zones. However, one might have expected greater involvement of the pIH, as extensive work has linked ventral hippocampal regions with processing rewards information. For instance, Nguyen et al. (2024) showed that ventral CA1 input to the nucleus accumbens is necessary for associating rewards with context. Similarly, Piquet et al. (2024) showed that inhibition of the ventral hippocampus impairs sensitivity to a degrading rewards value. This study also demonstrated that ventral hippocampal projections to medial prefrontal cortex are needed for appropriate responding to changing rewards value, as well as associating context and response outcome. Recent work in humans (Elliott et al., 2024) also shows that the dopaminergic midbrain, which is heavily involved in rewards processing, is more extensively connected to the anterior (ventral) hippocampus compared to the posterior (dorsal) hippocampus. Therefore, while our study focused on aversive learning, some changes in activity across these dorsal-intermediate subregions may be due to rewards expectation.

Taken together, our results show that remapping to a change in emotional context occurs across the dorsal-intermediate axis of the hippocampus. The DH showed greater remapping than the aIH and pIH, perhaps indicating that it is more involved in spatial emotional processing than previously thought. However, it is important to consider that, because animals in this study were overtrained on the behavioral task, possible dynamics between subregions during early learning could not be discerned (e.g., Komorowski et al., 2013). Our results also suggest that there may be differences within the intermediate hippocampus, with the aIH more involved in processing changes to the tone. Differences may also exist in how these subregions anticipate an incoming aversive conditioned stimulus, with the DH and aIH showing a change in activity and the pIH remaining stable. These results emphasize the fact that the current division of the dorsoventral axis into three regions may inadvertently miss possible important distinctive functional domains. These smaller functional domains may have nuanced roles in information processing. Lastly, these results also have implications for anxiety and stress-related disorders. Further work dissociating the exact roles of these intermediate subfields could provide insight for treatment of such disorders.

## Data Availability

The raw data supporting the conclusions of this article will be made available by the authors, without undue reservation.
