# Supplementary figures and images for: Differences in firing patterns along the dorsal-intermediate hippocampal axis in a fixed route during a change in emotional context
Source: Front Syst Neurosci. 2025 Nov 11;19:1632849. doi: 10.3389/fnsys.2025.1632849 (PMC12644059; doi:10.3389/fnsys.2025.1632849)

## Slide 1
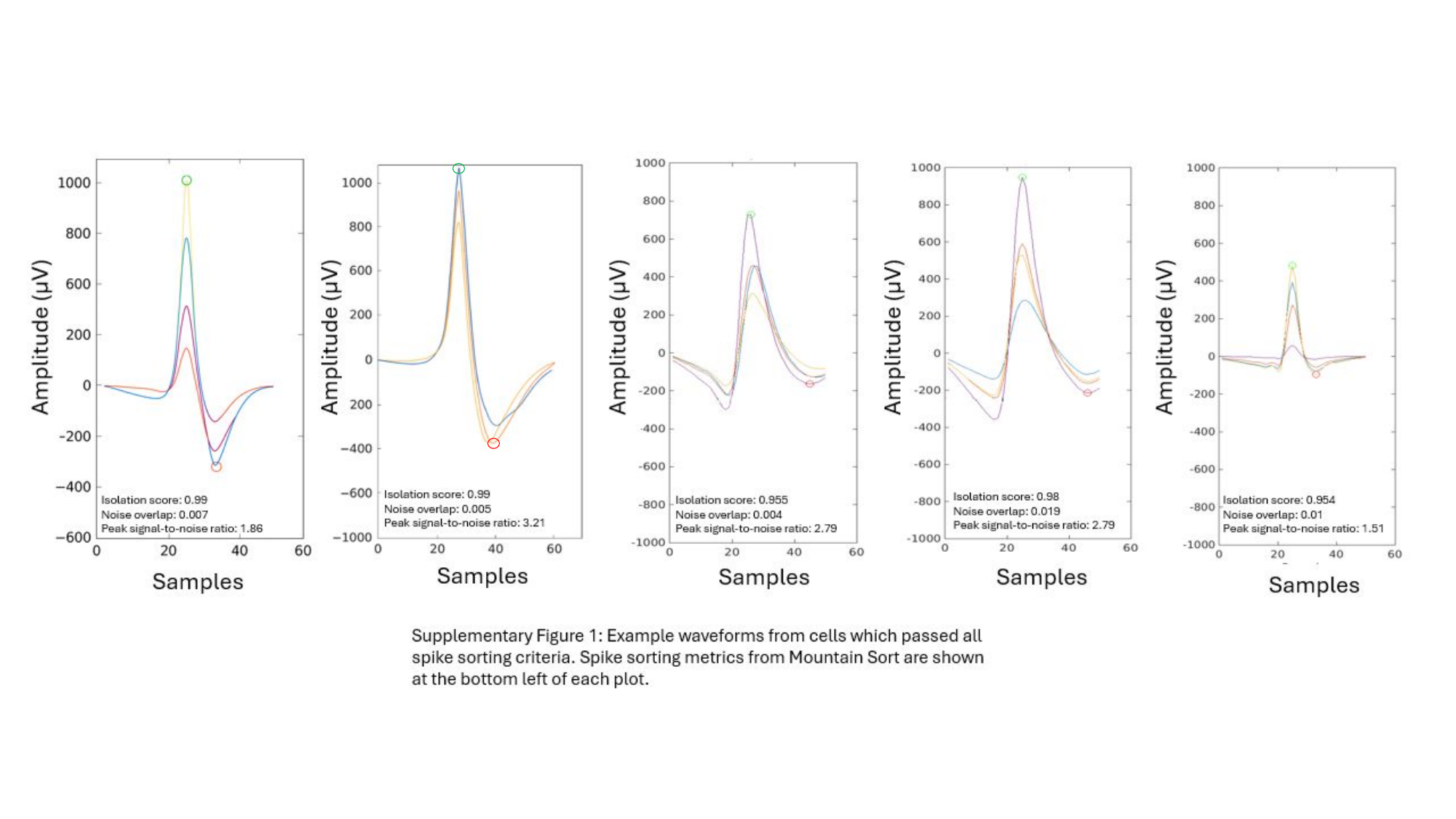

Supplement: Supplementary file 1 [file Presentation_1.pptx]
